# COVID-19 infection rate and mortality in a local health authority in Italy: Differences between home-dwelling and residential older adults

**DOI:** 10.1016/j.puhip.2023.100448

**Published:** 2023-11-04

**Authors:** Stefano Orlando, Carolina de Santo, Claudia Mosconi, Francesca Di Gaspare, Pelagia Chatzichristou, Leonardo Emberti Gialloreti, Fausto Ciccacci, Laura Morciano, Donatella Varrenti, Giuseppe Liotta, Leonardo Palombi

**Affiliations:** a Department of Biomedicine and Prevention, University of Rome Tor Vergata, Rome, Italy; b Local Health Authority - Rome 6, Department of Prevention, Service of Hygiene and Public Health, Rome, Italy; c UniCamillus, Saint Camillus International University of Health Sciences, Rome, Italy

**Keywords:** COVID-19, Elderly people, Long term care, Mortality rate, Risk of infection

## Abstract

**Objectives:**

The health emergency following the COVID-19 pandemic has seen hospital structures collapse and put in crisis nursing homes and other long-term care facilities worldwide. Our study aims to analyze and comparing the data relating to the infection rate and mortality for COVID-19 in the elderly over 75 living in the long-term care facilities and in the home-dwelling population.

**Study design:**

The study adopts a retrospective cohort design and was conducted in Italy, in the Lazio region, in the area of the Local Health Authority (LHA) named “Azienda Sanitaria Locale Roma 6”.

**Methods:**

Data were extracted from the COVID-19 surveillance system of the Lazio region. The primary outcome is the SARS-CoV-2 incidence rate in the period between 1^st^ September 2020 and 31^st^ May 2021. The secondary outcome is the mortality rate.

**Results:**

Living in a residential versus a home-dwelling setting was associated with a higher infection rate (OR 5.03, CI 4.67–5.43; p < 0.001). The mortality rate was higher for individuals living in a residential setting (19.3 %, CI 17.1%–21.7 %) than those living at home (13.0 %, CI 11.7%–14.5 %).

**Conclusions:**

These findings confirm the high mortality in Long-Term Care Facilities and provide new information on the infection rate. The containment measures adopted in the Long-Term Care Facilities during the COVID-19 pandemic, show limited correlation with reduced risk of contagion, but could have created unintended harm for the residents by increasing the social isolation and all other causes of mortality.

## 1 Introduction

In late 2019, an outbreak of severe atypical pneumonia occurred in Wuhan, Hubei Province, China. The causative agent was subsequently identified as a novel coronavirus, termed Severe Acute Respiratory Syndrome Coronavirus 2 (SARS-CoV-2). On February 11, 2020, the World Health Organization (WHO) officially named the disease COVID-19, an acronym for "Coronavirus Disease 2019." In response to the widespread dissemination of SARS-CoV-2, the WHO declared COVID-19 a global pandemic on March 11, 2020 [1].

In the first 2 years of the pandemic, SARS-CoV-2 has infected over 520 million people and resulted in more than 6.2 million deaths globally [2]. This unprecedented public health crisis has strained healthcare systems worldwide, leading to the collapse of medical facilities in numerous contexts. Long-term-care facilities (LTCFs) have been disproportionately affected, with consistent reports of elevated infection rates among their elderly residents [3,4]. Individuals in healthcare settings are often vulnerable due to preexisting comorbidities and cognitive impairments, complicating their ability to manage routine daily activities [5]. Consequently, they face an elevated risk of severe SARS-CoV-2 infection and higher mortality rates [6]. Compared to the general population, this demographic exhibits increased mortality from SARS-CoV-2 infection, associated with a higher prevalence of chronic diseases and comorbidities [7].

Consequently, the majority of research indicates an elevated mortality rate among elderly residents in nursing homes [4,5,7,8]. For instance, a recent study analyzing a cohort of 351 LTCFs in the United States eported a mortality rate of 21 % [5]. Another study from the United States suggested that the data concerning COVID-19 infections in LTCFs are likely underreported, describing the situation in nursing homes as a "perfect storm" [9]. Additionally, a European report indicated that the proportion of deaths attributable to SARS-Cov-2 among LTCF residents ranged between 31 % and 66 % [10].

Several studies have shed light on the situation in Italy, where 83 % of COVID-19-related deaths have occurred among individuals aged 70 and above. The average age of the deceased was 79 for men and 83 for women [11], underscoring the elevated vulnerability and higher risk of COVID-19 infection and mortality within the elderly demographic [12,13]. [[11], [12], [13]] The communal living arrangements in long-term care facilities (LTCFs), coupled with crowded common areas and challenges in isolating visitors, have facilitated more extensive spread of the SARS-CoV-2 virus within LTCFs compared to other healthcare settings. This is particularly significant given the limited resources and staff in LTCFs compared to hospitals [14,15]. However, there is a noticeable gap in the literature regarding specific analyses of SARS-CoV-2 infection incidence in residential settings.

Mortality rates are likely underestimated both globally and in Italy, and as of now, there are no official estimates concerning the rate of infection and death in residential facilities [3,13]. Moreover, current literature lacks data on mortality rates adjustedfor sex and age in two specific groups: residents of LTCFs who are 75 years old and above, and those who are home-dwelling. Most notably, there is an absence of data on COVID-19 incidence rates. The aim of our study is to examine the incidence of COVID-19 infection and associated mortality rates, in the population aged 75 and above, residing within the jurisdiction of the Local Health Authority (LHA) [in Italian: Azienda Sanitaria Locale (ASL)] denominated RM6, in Italy. Our analisys compares the LTCF population with home-dwelling population. Accurate data on infection incidence within nursing homes are crucial for enhancing preventive measures against future epidemics or pandemics, particularly to protect this highly vulnerable demographic.

## 2 Methods

The study was carried out in Italy, specifically in the Lazio region, within the jurisdiction of the LHA known as “ASL Roma 6”. The LHA serves as the administrative entity responsible for implementing the services of Italy's National Health System within a designated area. The particular jurisdiction covers the southern part of the Province of Rome, spanning an area of 756.84 Km^2^. As of January 1, 2019, Lazio is the second most populous region in Italy, with 5,879,082 residents. The region encompasses an area of 17,203 km^2^. Notably, 11.1 % of the total population is aged 75 or older. The age structure of the population in this region is consistent with the national demographic profile [16].

The study employed a retrospective cohort design, focusing on individuals aged 75 and above who tested positive for COVID-19 and received healthcare within the jurisdiction of the LHA Roma 6. The cohort also included individuals meeting the same criteria but resiled in LTCFs within the same area. Our study encompasses all reported cases from September 1, 2020, to May 31, 2021. This timeframe corresponds to what is commonly referred to as the "second wave" of the COVID-19 pandemic in Italy, a period marked by a significant rise in case numbers following a substantial decline during the summer months (May–August 2020). Importantly, the pandemic had been ongoing for six months prior to this, allowing ample time for the implementation of containment measures. Within the jurisdiction of LHA Roma 6, there were 559,750 residents, of which 53,980 were aged 75 75 or older. The proportion of older adults is represented by an old-age index of 150 [17], which is slightly lower than the index of the Lazio region (172.9) and the national index (182.6) [16].

Data for this study were sourced from the Lazio region's CoronaVirus Emergency Platform (CEP), a leading surveillance tool for monitoring individuals affected by the COVID-19 pandemic. The platform houses information on patients' clinical status and subsequent health outcomes following a confirmed SARS-Cov-2 diagnosis. Data entry was managed by a specialized team known as “Equipe Anti COVID-19”, who were also responsible for daily case notification on the regional CEP surveillance system. Additionally, the CEP platform facilitates the automated uploading of swab results from regional laboratories. These results were downloaded daily, and patients who tested positive for SARS-CoV-2 via real-time Reverse Transcription-Polymerase Chain Reaction (RT-PCR), were subsequently contacted by medical personnel from the LHA for epidemiological investigation. Following this, the team updated the patient information on a specialized “LHA COVID platform” – an IT platform designed for surveillance, management and control activities. This platform accesses and integrates data from the CEP platform, supplementing it with additional details reported by the contacted patients. Specifically, the healthcare team verified the living settings of the patients, determining whether they were residential or home-dwelling.

For the purposes of this study, two researchers affiliated with the LHA curated a database by extracting records of confirmed SARS-CoV-2 cases from the LHA COVID platform. The dataset was limited to include only individuals aged 75 and above who resided within the jurisdiction of the LHA. A case was defined as an individual who tested positive for SARS-CoV-2 nucleic acid using the real-time RT-PCR method. Subsequently, this dataset was enriched by merging it with additional information related to the health outcomes of the infection. This included details on patients hospitalization and any transfers to other LHAs, as captured in the regional CEP platform.

The primary outcome of interest in this study is the incidence rate of SARS-CoV-2 infections during the observation period. The numerator for this rate consists of the total number of individuals infected during the period, while the denominator comprises the number of individuals in both the home-dwelling and residential settings. The size of the home-dwelling population was determined based on the total number of individuals aged 75 and over in the LHA area for the year 2020, as reported in the Open Salute Lazio health data portal [17]. The number of residents in LTCFs was calculated from the total number of available beds in specific types of facilities – namely, nursing homes, assisted-living facilities, rest homes and LTCFs that exclusively accommodated older adults. Facilities catering to the general population, children, or those operating under a semi-residential scheme were excluded from consideration. The secondary outcome under study was the mortality rate. Additional covariates, such as age, sex, and the rate of hospitalization due to SARS-CoV-2 infection, were also taken into account in the analysis.

During the data collection phase, the national anti-COVID vaccination campaign was initiated. As per the national vaccination plan, older adults residing in LTCFs were prioritized for vaccination alongside healthcare workers. However, the actual vaccination date for this demographic varied due to organizational and logistical challenges. Consequently, we designated December 27th, 2020, as the commencement date of the vaccination campaign for LTCF residents in our study. Our descriptive statistics also delineate the number of patients who contracted the SARS-CoV-2 infection both before and after the onset of the vaccination campaign.

Statistical analyses were conducted using R software, version 4.1.1 [18] (packages: gtsummary [19], epitools [20]). In term of descriptive statistics, we presented percentages for categorical variables such as sex, hospitalization status, and the period of infection relative to the initiation of the vaccination campaign. These differences were evaluated using the chi-squared test. For age, which is a continuous variable, we reported the mean and standard deviation, and employed the Wilcoxon rank-sum test for comparisons. The odds ratio for COVID-19 infection among residents of LTCFs compared to home-dwelling population was calculated, along with a 95 % confidence interval. Furthermore, we conducted a binary logistic regression to assess the mortality risk between the two groups and a multivariable regression to determine the risk adjusted for age and sex.

## 3 Results

The study encompassed 3585 individuals who received a positive SARS-CoV-2 test result, as reported to the Local Health Authority (LHA). This constitutes 6.58 % of the total LHA population. Of these individuals who tested positive, 2410 were home-dwelling, making up 4.9 % of the home-dwelling population. Meanwhile 1175 wereresidents of LTCFs, accounting for 20.7 % of the LTCF resident population. Table 1 provides a detailed breakdown of the sample, including age, sex, rates of hospital admission, and infection period.

**Table 1:** Descriptive statistics.

Characteristic	Home-dwelling, N = 2,410^a^	Residential, N = 1,175^a^	p-value^b^
**Age**	82.3 (5.5)	86.8 (5.9)	<0.001
**Sex**			<0.001
*Men*	1027 (42.6 %)	280 (23.8 %)	
*Women*	1383 (57.4 %)	895 (76.2 %)	
**Admitted to hospital**	682 (28.3 %)	362 (32.2 %)	0.020
*Missing*	4	50	
**Period of infection**			0.004
*After vaccination campaign onset*	675 (28.0 %)	276 (23.5 %)	
*Before vaccination campaign onset*	1735 (72.0 %)	899 (76.5 %)	

a Mean (SD); n (%).

b Wilcoxon rank sum test; Pearson's Chi-squared test.

Residing in a LTCF, as opposed to a home-dwelling environment, was correlated with a higher rate of infection with an odds ratio (OR) of 5.03, and a 95 % confidence interval (CI) of 4.67–5.43 (p < 0.001). Additionally, the mortality rate was elevated for individuals in residential settings at 19.3 % (CI 17.1%–21.7 %), compared to 13.0 %, (CI 11.7%–14.5 %) for those living at home. Table 2 presents both binary and a multivariable logistic regression models. The unadjusted OR for mortality among individuals in residential settings was 1.60 (CI 1.32–1.93, p < 0.001) which adjusted to 1.43 (CI 1.16–1.76, p = 0.001) when accounting for age and sex.

**Table 2:** Logistic regression (binary and multivariable), Living setting and death.

Characteristic	Univariate analysis			Multivariable analysis		
	OR^a^	95 % CI^a^	p-value	OR^a^	95 % CI^a^	p-value
Group						
*Home-dwelling*	–	–		–	–	
*Residential*	1.60	1.32, 1.93	<0.001	1.43	1.16, 1.76	<0.001
**Age**				**1.07**	**1.06, 1.09**	**<0.001**
**Sex**						
*Men*				–	–	
*Women*				0.40	0.33, 0.49	<0.001

a OR = Odds Ratio, CI = Confidence Interval.

## 4 Discussion

The most relevant salient insight from our study is the significantly elevated risk of infection in LTCFs as compared to individuals living at home (OR 5.03). While our findings corroborate the well-documented higher mortality rates in LTCFs within existing Italian scientific literature [[6], [7], [8],^10^,[21], [22], [23], [24], [25]], they also introduce new data concerning infection risk across different living settings. While elevated mortality rates in LTCFs are not the primary focus of this study, it's crucial to acknowledge that various factors may contribute to this disparity. Existing research suggests that LTCF residents may have a higher prevalence of comorbidities and may be subject to polypharmacy, both of which could influence mortality rates [5]. Additionally, the absence of familial support could impact immune response and, consequently, mortality [9,25].

The issue of heightened infection risk within LTCFs has frequently been covered in media reports: however, to our knowledge, there have been no rigorous studies that offer a comparative analysis similar to our own. Existing studies predominantly focus on identifying the potential causes for the elevatedmortality rates from COVID in LTCFs often attributing it to the frailty of the residents, or the limitations of the care setting, such as staffing constraints and confined communal spaces. These studies, however, do not provide a comprehensive analysis of the infection rate itself. There is only one study that has compared COVID-related mortality in LTCFs residents to that in the general population, examining data from 12 OECD member countries [6]. Yet, it's worth noting that theseare not the results of epidemiological research but rather estimations made by national statistical offices. As such, they are based on partial samples or rough estimates, a point explicitly acknowledged by the authors when discussing data from Italy and Spain.

Despite the implementation of stringent containment measures by Italian public health authorities since the onset of the pandemic, a notably higher rate of infection has been observed in LTCFs. These measures included prohibiting visits from family members and friends, as well as mandating the use of personal protective equipment (PPE) by both residents and staff to mitigate virus transmission [26].

Our findings prompt us to consider two significant factors. The first pertains to the possible inadequacy or incomplete implementation of containment measures within LTCFs. The second factor involves the inherent health risks associated with residing in such facilities. While the containment measures in LTCFs, were designed to mitigate infection risks, they inadvertently harmed residents by exacerbating social isolation, which is also linked to other mortality causes [[27], [28], [29], [30], [31], [32]]. For instance, restricting family members and other visitors from accessing LTCFs proved to be a counterproductive measure. Regarding the second factor, although LTCFs are generally recommended for patients with chronic, multiple comorbidities who require substantial care, many of these individuals could feasibly be managed at home through community healthcare services or in hospitals for acute conditions [4,5]. The heightened vulnerability of frail patients to infectious diseases has been starkly revealed during this pandemic, putting them at elevated risk for COVID-19-related mortality. Therefore, it is crucial to allocate these patients to the most appropriate care setting to minimize exposure to risk factors that could worsen their overall health. This could include restricting LTCF admissions to only those older adults who truly require such specialized care.

Our study's findings align with the objectives set forth inthe “National Recovery and Resilience Plan” (PNRR), issued by Italian government in 2021 [33]. Specifically, within "Mission 6: Health", the plan outlines a five-year strategy and allocate substantial resources to enhance home-based and community healthcare services. This is aimed at efficiently managing the growing number of patients with chronic conditions and multiple comorbidities, thereby reducing inappropriate hospital admissions and minimizing exposure to suboptimal care settings.

One of the primary strengths of our study lies in the comprehensiveness of the cohort, which encompasses all individuals who tested positive for SARS-CoV-2 within the jurisdiction of the LHA. This extensive data set enhances the robustness of our findings and their implications for healthcare policy.

Among the limitations of our study, several are inherent to its design. Our cohort only included patients who tested positive for the molecular test for SARS-CoV-2 via molecular testing, introducing a selection bias. Specifically, some individuals, particularly those who were asymptomatic or exhibited mild symptoms, may never have undergone such testing. This bias could be more pronounced among the home-dwelling population. In contrast, residents of LTCFs were routinely tested for preventive reasons whenever a case was reported within their facility, even if they were asymptomatic. This practice may have led to an underestimation of SARS-CoV-2 cases among home-dwellers. Another limitation pertains to the generalizability of our findings. The data collected are specific to one Local Health Authority (LHA) in the Lazio region, which may not be representative of the situation across Italy. Lastly, our database lacked information on patient comorbidities, restricting our ability to adjust mortality outcomes for this crucial confounding variable. Despite these limitations, the study offers valuable insights into the disparate rates of SARS-CoV-2 infection and associated mortality among different living settings.

The COVID-19 pandemic has exerted profound effects on populations residing in LTCFs globally. Its impact is evident not merely in elevated mortality rate, surpassing those of previous years for the same demographic, but also, as our study elucidates, in the high incidence of infections within this particular setting. These results have multiple implications. First, there is an urgent need to evaluate and scrutinize the efficacy of existing containment measures implemented in LTCFs, particularly those that exacerbate social isolation among residents. Second, it is imperative to enhance the standard of care for frail and multi-morbid individuals to mitigate the risk of infection from communicable diseases. Consequently, careful consideration should be given to selecting the most appropriate care setting for these vulnerable populations, with an emphasis on increasing access to community-based care models, such as home care, as a preferable alternative. We advocate for additional studies on a larger scale, optimally at a national level, to corroborate and expand upon these findings.

## Conflicts of interest

We have no conflicts of interest to disclose. All authors declare that the research protocol has been approved by the Ethics Committee of the University of Tor Vergata and it conforms with the provisions of the Helsinky Declaration (as revised in Tokyo 2004).

## Funding

This research did not receive any specific grant from funding agencies in the public, commercial, or not-for-profit sectors.

## Declaration of competing interest

The authors declare that they have no known competing financial interests or personal relationships that could have appeared to influence the work reported in this paper.

## References

[1] WHO (2020). Pneumonia of unknown cause – China. https://www.who.int/emergencies/disease-outbreak-news/item/2020-DON229.

[2] WHO coronavirus (COVID-19) dashboard. https://covid19.who.int.

[3] Sepulveda E.R., Stall N.M., Sinha S.K. (2020 Nov). A comparison of COVID-19 mortality rates among long-term care residents in 12 OECD countries. J. Am. Med. Dir. Assoc..

[4] Graham N.S.N., Junghans C., Downes R., Sendall C., Lai H., McKirdy A. (2020 Sep 1). SARS-CoV-2 infection, clinical features and outcome of COVID-19 in United Kingdom nursing homes. J. Infect..

[5] Panagiotou O.A., Kosar C.M., White E.M., Bantis L.E., Yang X., Santostefano C.M. (2021 Apr 1). Risk factors associated with all-cause 30-day mortality in nursing home residents with COVID-19. JAMA Intern. Med..

[6] Sepulveda E.R., Stall N.M., Sinha S.K. (2020 Nov). A comparison of COVID-19 mortality rates among long-term care residents in 12 OECD countries. J. Am. Med. Dir. Assoc..

[7] Mccann M., O’reilly D., Cardwell C. (2009).

[8] Canouï-Poitrine F., Rachas A., Thomas M., Carcaillon-Bentata L., Fontaine R., Gavazzi G. (2021 Sep 1). Magnitude, change over time, demographic characteristics and geographic distribution of excess deaths among nursing home residents during the first wave of COVID-19 in France: a nationwide cohort study. Age Ageing.

[9] Ouslander J.G., Grabowski D.C. (2020 Oct 1). COVID-19 in nursing homes: calming the perfect storm. J. Am. Geriatr. Soc..

[10] Miralles O., Sanchez-Rodriguez D., Marco E., Annweiler C., Baztan A., Betancor É. (2021 Feb). Unmet needs, health policies, and actions during the COVID-19 pandemic: a report from six European countries. Eur Geriatr Med.

[11] Abbatecola A.M., Antonelli-Incalzi R. (2020 May 1). COVID-19 spiraling of frailty in older Italian patients. J. Nutr. Health Aging.

[12] Superiore di Sanità I., Giacomozzi C., Donfrancesco C., Lo Noce C., Bacigalupo I., Fortunato P.D. (2020).

[13] Ventura F., Molinelli A., Barranco R. (2021 May 1). COVID-19-related deaths in residential care homes for elderly: the situation in Italy. J. Forensic Leg. Med..

[14] Falcone M., Russo A., Gentiloni Silverj F., Marzorati D., Bagarolo R., Monti M. (2018). Predictors of mortality in nursing-home residents with pneumonia: a multicentre study. Clin. Microbiol. Infection.

[15] McMichael T.M., Clark S., Pogosjans S., Kay M., Lewis J., Baer A. (2020). COVID-19 in a long-term care facility - king county, Washington, february 27-march 9, 2020. MMWR Morb. Mortal. Wkly. Rep..

[16] ISTAT No title. http://dati.istat.it.

[17] REGIONE LAZIO (2020). Open salute lazio. https://www.opensalutelazio.it/salute/.

[18] R Core Team. R (2021).

[19] Sjoberg D., Whiting K., Curry M., Lavery J., Larmarange J. (2021). Reproducible summary tables with the gtsummary package. R J.

[20] Aragon T.J. (2020).

[21] Ventura F., Molinelli A., Barranco R. (2021 May 1). COVID-19-related deaths in residential care homes for elderly: the situation in Italy. J. Forensic Leg. Med..

[22] Panagiotou O.A., Kosar C.M., White E.M., Bantis L.E., Yang X., Santostefano C.M. (2021 Apr 1). Risk factors associated with all-cause 30-day mortality in nursing home residents with COVID-19. JAMA Intern. Med..

[23] Ouslander J.G., Grabowski D.C. (2020 Oct). COVID-19 in nursing homes: calming the perfect storm. J. Am. Geriatr. Soc..

[24] Graham N.S.N., Junghans C., Downes R., Sendall C., Lai H., McKirdy A. (2020 Sep 1). SARS-CoV-2 infection, clinical features and outcome of COVID-19 in United Kingdom nursing homes. J. Infect..

[25] Yeh T.C., Huang H.C., Yeh T.Y., Huang W.T., Huang H.C., Chang Y.M. (2020 Oct). Family members' concerns about relatives in long-term care facilities: acceptance of visiting restriction policy amid the COVID-19 pandemic. Geriatr. Gerontol. Int..

[26] Talic S., Shah S., Wild H., Gasevic D., Maharaj A., Ademi Z. (2021 Nov 18). Effectiveness of public health measures in reducing the incidence of covid-19, SARS-CoV-2 transmission, and covid-19 mortality: systematic review and meta-analysis. BMJ.

[27] Unruh M.A., Yun H., Zhang Y., Braun R.T., Jung H.Y. (2020 Jul). Nursing home characteristics associated with COVID-19 deaths in Connecticut, New Jersey, and New York. J. Am. Med. Dir. Assoc..

[28] Ruopp M.D. (2020 Jul). Overcoming the challenge of family separation from nursing home residents during COVID-19. J. Am. Med. Dir. Assoc..

[29] O'Caoimh R., O'Donovan M.R., Monahan M.P., Dalton O'Connor C., Buckley C., Kilty C. (2020 Oct 26). Psychosocial impact of COVID-19 nursing home restrictions on visitors of residents with cognitive impairment: a cross-sectional study as part of the engaging remotely in care (ERiC) project. Front. Psychiatr..

[30] Abbasi J. (2020 Aug 18). Social isolation—the other COVID-19 threat in nursing homes. JAMA.

[31] Szczerbińska K. (2020 Aug). Could we have done better with COVID-19 in nursing homes?. Eur Geriatr Med.

[32] Chu C.H., Donato-Woodger S., Dainton C.J. (2020 Oct). Competing crises: COVID-19 countermeasures and social isolation among older adults in long-term care. J. Adv. Nurs..

[33] (2021). Piano Nazionale di Ripresa e Resilienza (PNRR). https://www.governo.it/it/articolo/piano-nazionale-di-ripresa-e-resilienza/16782.

